# Two Cannulated Screws Provide Sufficient Biomechanical Strength for Prophylactic Fixation in Adult Patients With an Aggressive Benign Femoral Neck Lesion

**DOI:** 10.3389/fbioe.2022.891338

**Published:** 2022-07-07

**Authors:** Guangtao Fu, Guoqing Zhong, Zehong Yang, Shi Cheng, Limin Ma, Yu Zhang

**Affiliations:** ^1^ Department of Orthopedics, Guangdong Provincial People’s Hospital, Guangdong Academy of Medical Sciences, Guangzhou, China; ^2^ Shantou University Medical Colleges, Shantou, China; ^3^ Department of Radiology, Sun Yat-Sen Memorial Hospital, Sun Yat-Sen University, Guangzhou, China

**Keywords:** femoral neck, benign lesion, cannulated screw, prophylactic fixation, biomechanical analysis

## Abstract

**Background:** Two cannulated screws were proposed for prophylactic fixation in adult patients with an aggressive benign femoral neck lesion in recent literature. However, the biomechanical properties of this intervention have not yet been investigated.

**Methods:** After the evaluation of the heterogeneity of bone mineral density and geometry *via* quantitative computed tomography, 24 embalmed adult human cadaver femurs were randomized into the control, inferior half of the anterior cortical (25%) bone defect, entire anterior cortical (50%) bone defect, and the 50% bone defect and two cannulated screw group. Biomechanical analysis was conducted to compare the stiffness and failure load among the four groups when mimicking a one-legged stance. A CT-based finite element analysis (FEA) was performed to mimic the cortical and cancellous bone defect and the implantation of two cannulated screws of the four groups. Measurements of the maximal displacement and von Mises stress were conducted with the longitudinal load force and boundary conditions being established for a one-leg-standing status.

**Results:** We noted a significant improvement in the failure load after the insertion of two 6.5 mm cannulated screws in femurs with 50% bone defect (+95%, *p* = 0.048), and no significant difference was found between the screw group and the intact femur. Similar trends were also found in the measurements of stiffness (+23%, *p* > 0.05) *via* biomechanical testing and the von Mises stresses (−71%, *p* = 0.043) by FEA when comparing the screw group and the 50% bone defect group.

**Conclusion:** Our findings suggest that two cannulated screws provided sufficient biomechanical strength for prophylactic fixation in adult patients with an aggressive benign femoral neck lesion even when the entire anterior cortical bone is involved.

## Introduction

The femur neck is one of the most common anatomical sites of benign and tumor-like bone lesions (Shin et al., 2013). Although those lesions in the femur neck are most commonly detected incidentally and asymptomatically, a high degree of concern is raised for pathological fracture due to loss of normal anatomical structure and less residual bone stock in this weight-bearing site (Shi et al., 2021). Furthermore, the economic and clinical value of prophylactic stabilization when performed on patients with painful lesions compromising the structural integrity of long bones has been well-proven (Blank et al., 2016). Thus, prophylactic internal fixation is currently preferred for aggressive benign femoral neck lesions in adults (Nakamura et al., 2015; Panchwagh et al., 2018). Several options of the internal implant for an aggressive benign femoral neck lesion were previously reported, including cannulated screws (Singh et al., 2015; Erol et al., 2016), intramedullary fixation (Zhang et al., 2017), and compression hip screw (Nakamura et al., 2015). However, there is still no agreed consensus on the optimal selection of the internal fixation implant which provides not only sufficient biomechanical strength but also a minimally invasive approach after curettage (Shi et al., 2021).

The insertion of three cannulated screws is a well-proven treatment for adult non-displaced femoral neck fractures (Filipov, 2019). However, the principal blood supply sources for the femoral head, the epiphyseal arterial network system (Zhao et al., 2017), are easily damaged when placing the third screw in the superior and posterior area, and this complication is difficult to evaluate using perioperative radiographs (Hoffmann et al., 2019). It was also reported that the first two cannulated screws provide not only sufficient biomechanical stability but also less trauma and lower risk (5% vs. 7%) of femoral head avascular necrosis in adult patients with non-displaced femoral neck fractures when compared with the traditional three cannulated screws (Krastman et al., 2006; Gupta et al., 2016; Widhalm et al., 2019). Consequently, prophylactic fixation with two cannulated screws was recently proposed by some surgeons for the treatment of aggressive benign lesions in the femoral neck due to the earlier mentioned benefits (Singh et al., 2015; Erol et al., 2016). But, the biomechanical properties of this intervention have not yet been investigated, although satisfied short-term clinical outcomes were reported in a limited sample size.

Thus, the present study aims to evaluate the biomechanical stability of using two 6.5 mm cannulated screws as prophylactic fixation for an aggressive benign femoral neck lesion *via* cadaveric biomechanical testing and finite element analysis.

## Methods and Materials

### Specimen Preparation

Twenty-four embalmed adult human cadaver femurs were obtained from the Department of Anatomy, Southern Medical University in Guangzhou, China (average age: 77.8 years, range: 67–89 years; 15 males and nine females). The Institutional Review Board of Guangdong Provincial People’s Hospital waived the informed consent procedure for this portion of the study. After removing soft tissue, anterior–posterior and lateral radiographs were taken for each femur to exclude pre-existing disease, deformity, or trauma.

### Quantitative Computed Tomography Scanning

**FIGURE 1:**
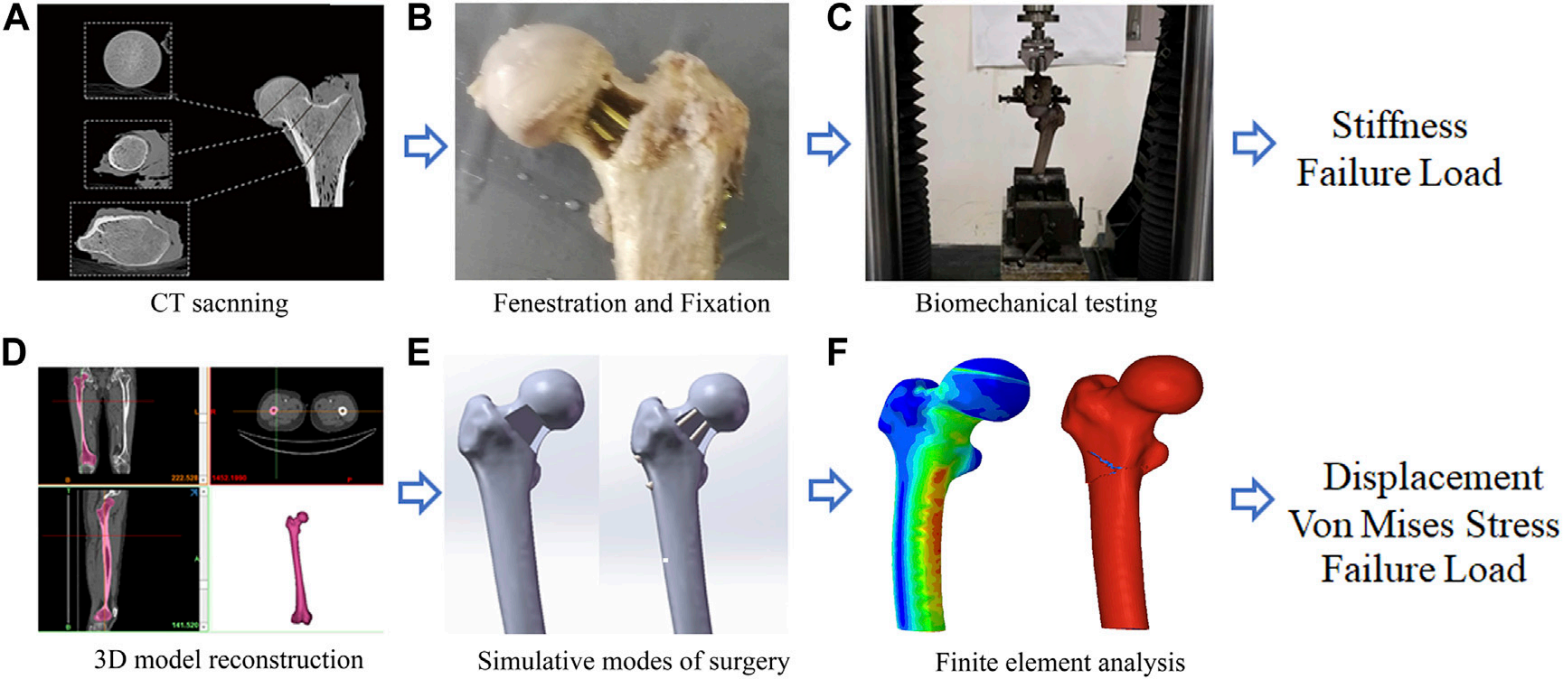
A graphical overview of the present study design. **(A)** CT scanning; **(B)** Fenestration and Fixation; **(C)** Biomechanical testing; **(D)** Three-Dimension model reconstruction; **(E)** Simulative modes of surgery; **(F)** Finite element analysis.

To evaluate the heterogeneity of bone density and geometry in the femur neck during the biomechanical testing, QCT scans were carried out using a clinical scanner (SIEMENS SOMATOM 64, 140 kV, 80 mAs, 0.5 * 0.5 mm/pixel resolution, and 1 mm slice thickness) for each cadaver femur. Using a calibration phantom (MINDWAYS Software, Inc., San Francisco, CA), grayscale values were mapped to K_2_HPO_4_ equivalent density (ρ_KHP_) using five tubes with reference densities and Hounsfield Units (HUs) were calibrated. Segmentation of the bone hard tissue from its surroundings was performed for each slice. The raw QCT images (in DICOM format) were converted into a binary format, and a combination of user-defined threshold limits with an edge following scheme was used for generation of the hard-tissue contours and elimination of soft tissue. BMD (g/cm^3^) of the femoral head, femoral neck, and intertrochanteric region was measured (Figure 1A).

Specific to geometric measurements, the femoral neck–shaft angle was defined as the angle formed between the femoral neck axis and the femoral shaft axis, and this angle was measured on QCT scout images for each patient (Lee et al., 2017). Hip axis length was defined as the length along the femoral neck axis, from the lateral aspect of the greater trochanter to the inner pelvic rim (Maeda et al., 2011). Neck width was also measured as described by Maeda et al. (2011).

### Biomechanical Testing

**FIGURE 2:**
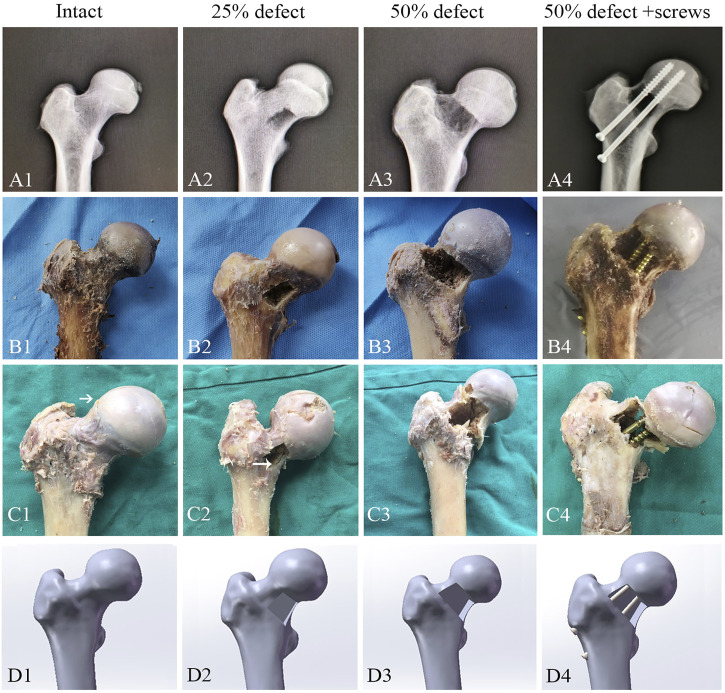
Representative images for X-ray **(A1–A4)**, cadaveric tests **(B1–B4)**, femoral neck fracture **(C1–C4)**, and FEA **(D1–D4)** of the control group, 25%-defect group, 50%-defect group, and 50%-defect + two cannulated screw group.

After QCT scanning, 24 specimens were randomly and equally allocated to the following four groups: control group, 25% defect group, 50% defect group, and 50% defect + screw group (Figures 2B1–B4). The area of the bone defect was defined according to the description outlined by Çaypınar et al. (2016). Briefly, the femur neck is most likely a cylinder, and we adopted a cylinder with a 10 cm base (the circumference of the circle) and a height of 7 cm. For example, a 25% bone defect was obtained when we create a rectangle anterior cortical bone defect using a bone drill, with a 2.5 cm base and a 7.0 cm height. In the 50% defect + screw group, two 6.5 mm AO cannulated screws were inserted in parallel into the coronal plane at a 125° angle from the femoral shaft over the guiding K-wires. The two cannulated screws were inserted close to the superior and inferior margin of the femoral neck, and both screws advanced to within 5 mm of the subchondral bone to meet the requirements of the tip apex distance. The area of bone defect and the position of cannulated screws were further confirmed by X-ray (Figures 2A1–A4).

The specimens were then mounted with a fixed shaft in a servo-hydraulic test frame (ElectroForce 3500, TA instrument, United States). The angle between the loading axis and the proximal shaft was 15° during the mechanical testing to mimic the weight of the femur during a one-legged stance (Figure 1C) (Dou et al., 2019). The load was applied to the most cranial portion of the femoral head in the plane, spanned by the neck axis and the proximal femur axis. An axle bearing was inserted between the embedded femoral head and the test frame to allow rotation orthogonally to the loading axis through the femoral head. Before each test, all specimens were manually preloaded with a maximum of 100 N to avoid play between the embedded specimens and the test setup. The axial force was recorded *via* a 100-kN load cell (U3 force transducer; HBM, Darmstadt, Germany).

To keep the femora remained in the linear–elastic regime before the ultimate axial loading failure testing, a displacement range of 1 mm was chosen based on pre-tests, ensuring test conditions would not initiate plastic deformation and irreversible damage to the specimen. The axial stiffness was determined by vertically applying a vertical displacement of 1 mm maximum using displacement control 5 mm/min during the axial loading testing (Dou et al., 2019). Following a short relaxation period, a second compression cycle was applied with the same speed until the failure of load.

### Three-Dimensional Model Construction

For a more detailed evaluation of the biomechanical property of the two cannulated screws, we also performed FEA. A healthy volunteer (male, 30-year-old) without previous history of surgery, trauma, and deformity in the lower limbs was recruited. Computerized tomography (CT) scan (SIEMENS SOMATOM 64) was performed on the lower limbs of the volunteer, with the slice thickness set at 0.5 mm. Image data of the right femur were imported into Mimics (version 21.0, Materialise NV, Leuven, Belgium) for three-dimensional (3D) reconstruction, which was based on the gray value of the tissue and segmentation of the region (Figure 1D). Subsequently, the model in STL format was imported into the Geomagic Wrap (version 2017, Geomagic Corporation, United States) for smoothing, meshing, and fitting surface processing.

A 3D computer-aided design software Solidworks (version 2017, Dassault Systèmes, Waltham, MA, United States) was then used to create the cortical and cancellous bone defect caused by the lesion and mimic the implantation of two cannulated screws (Figure 1E). Four models including control (intact), 25% bone defect, 50% bone defect, and 50% bone defect + two 6.5 mm cannulated screws were developed (Figures 2D1–D4). The 25% and 50% bone defects were obtained as outlined previously . To simulate the characteristics of the surgery more accurately, the position of the screws was strictly followed as per conventions of clinical practice, which we described in detail in the biomechanical analysis section.

### Static Analysis

Data from the four models were imported into Abaqus 6.14 software (Dassault Systèmes S.A., France) to generate C3D10 tetrahedral elements. As per the study of Palumbo et al. (2014), Ti6Al-4V titanium was used as the internal fixation instruments, and the property parameters (Young’s Modulus and Poisson’s Ratio) of materials are listed in Table 1. The models were meshed to 1.0 mm, equal-sized facets, with >1,000,000 elements and ranging from 1,088,910 to 1,464,698. A mesh convergence test was conducted so that the deviation was less than 2%. In the current study, the cortical bone and cancellous bone interfaces were given with a tie feature. Regarding the interfaces between the screw thread and bone, the interfaces between the bone and metal were assigned as a sliding contact, with the frictional coefficient being 0.46 (Jiang et al., 2021).

**TABLE 1:** Material properties are applied for the static and fracture analysis of finite element models.

Material properties	Cortical bone	Cancellous bone	Ti6Al–4V titanium
Young’s modulus (E, MPa)	19,650	1,260	117,000
Poisson’s ratio (μ)	0.3	0.2	0.3
Apparent density (ρ, g/cm3)	1.525	0.433	4.5
Yield stress (σ, Mpa)	136.728	3.434	1086
Failure strain (%)	0.70%	0.70%	-

It was reported that during static balance, approximately one-third of a person’s body weight falls on each hip vertically (Çaypınar et al., 2016). Accordingly, a load of 700 N was applied straight down on the femoral head to simulate the one-leg-stance of an obese adult. Subsequently, the load force and boundary conditions were established for a one-leg-stance (Figure 1F). To mimic biomechanical testing, the area on the weight-bearing region of the femoral head, which intersects the mechanical axis, was set as the loading position, and the distal end of the femur was fixed. Measurements of von Mises stress were queried at 14 points of interest on the femoral neck that lay within the mid-coronal plane (Figure 5A). Von Mises stresses on the superior margin of the femoral neck were defined as the mean of von Mises stress from point 1 to point 7. The mean of von Mises stress from point 8 to point 14 was calculated as the Von Mises stresses on the inferior margin.

### Fracture Mechanics Analysis

In order to test the failure loads, four models were imported to Hypermesh14.0 software (Altair, United States) to be meshed as tetrahedral with a size of 2 mm^21^. The mimic of the femoral neck fracture was established by Hypermesh and LS-DYNA software. An elastic–plastic material model (*MAT_03) in Hypermesh14.0 was used to simulate cortical and trabecular bone behaviors. According to previous studies (Li et al., 2010; Khor et al., 2018), the femoral fracture was defined by detecting a failure strain that was initiated and propagated by element deletion as long as the strain of an element reaches the limit. The fracture analyses were performed by LS-DYNA (LSTC, Livermore, CA) software to investigate the biomechanical effect of the failure test (Zheng et al., 2021). Material properties applied for fracture analysis are shown in Table 1. The density of cortical bone, cancellous, and Ti6Al–4V titanium was obtained from previous reports (Dhanopia and Bhargava, 2017; Zheng et al., 2021). According to the equations between bone density (ρ), Young’s modulus (E), and yield stress (σ) (Morgan and Keaveny, 2001; Duchemin et al., 2008), cortical and cancellous bone’s yield stress was calculated respectively. The contact settings between bone and screws were the same as those in the static analysis. Meanwhile, the loading settings were applied according to the biomechanics test of the cadaveric femurs until the failure of load.

### Statistical Analysis

Continuous data were expressed as mean ± standard deviation or median with an interquartile range. Pearson’s correlation analysis was conducted to test the correlation between maximum displacements of FEA and the stiffness of the mechanical test. The data of QCT measurement, biomechanical test, and FEA analysis of different groups were compared with one-way ANOVA followed by the least significant difference (LSD) test for multiple comparisons. The two-sided Student’s t-test was used to compare the von Mises stresses of the inferior margin and the superior margin of the femoral neck within the groups. Bonferroni correction was performed for multiple testing. Differences were considered significant if *p* < 0.05. SPSS 20.0 statistical software (Chicago, IL, United States) was used for these analyses.

## Results

### Biomechanical Testing

**FIGURE 3:**
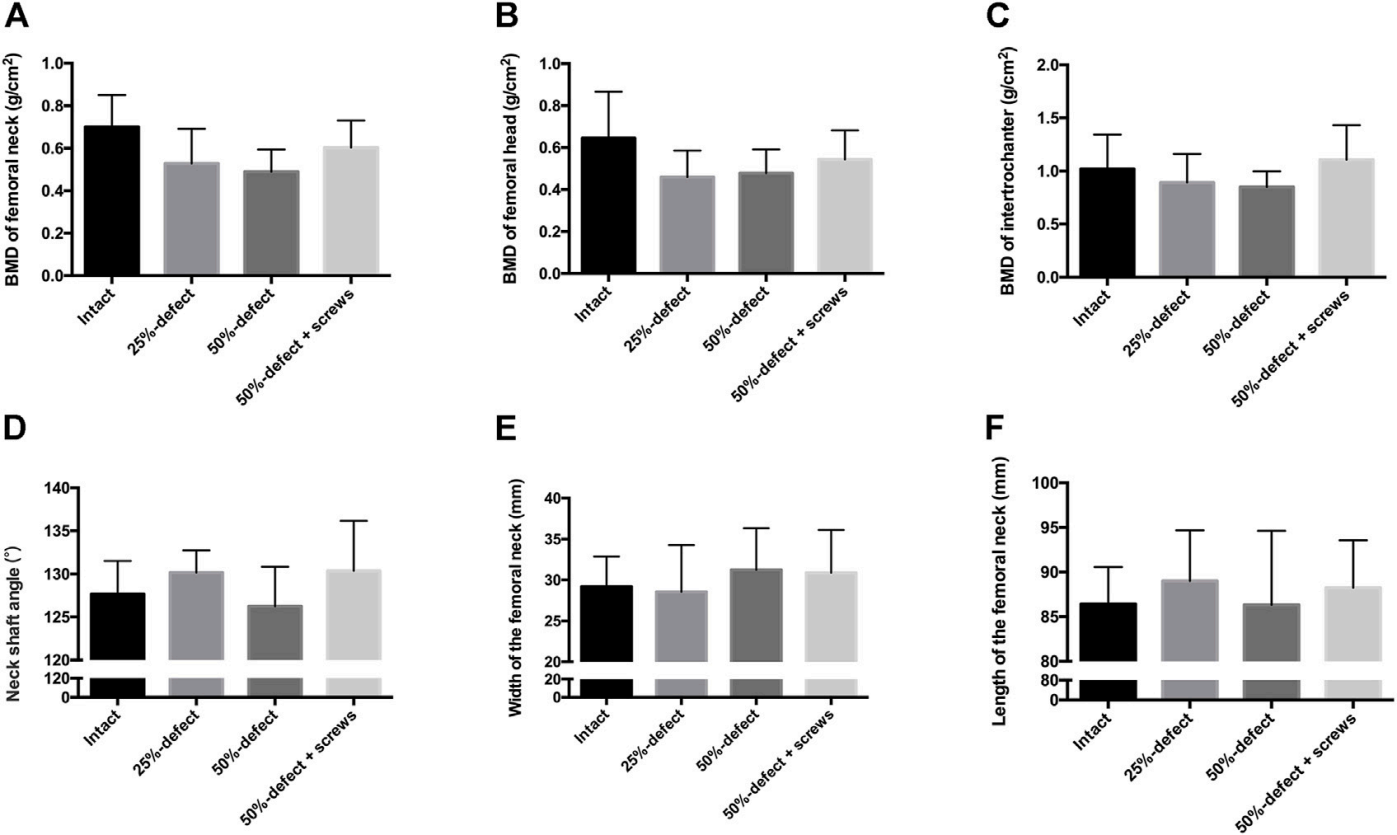
QCT-based BMD of each group was measured at the femoral neck **(A)**, femoral head **(B)**, and intertrochanteric region **(C)** of the cadaver femurs. Measurements of the femoral geometry parameters include neck-shaft angle **(D)**, width of the femoral neck **(E)**, and length of the femoral neck **(F)**. All the data are presented as mean + std dev.

No significant difference in BMD was measured at the femoral neck (Figure 3A), the femoral head (Figure 3B), or the intertrochanteric region (Figure 3C). Furthermore, no significant difference was observed between femoral geometry parameters including neck-shaft angle (Figure 3D), width of the femoral neck (Figure 3E), and length of the femoral neck (Figure 3F) of the cadaver femurs within the four groups. Thus, any potential bias caused by heterogeneity of bone density and geometry in the femur neck during the biomechanical testing is likely limited.

**FIGURE 4:**
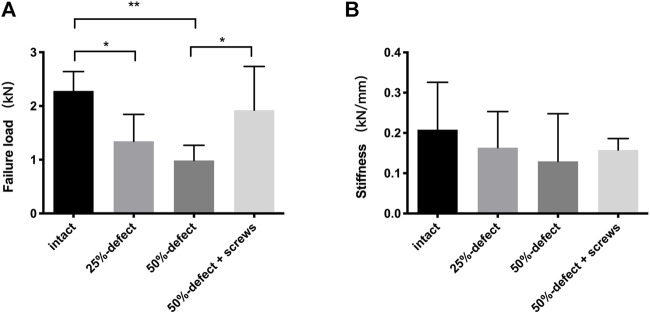
Comparisons of failure load **(A)** and stiffness **(B)** within the four groups. * indicated *p* < 0.05, ** indicated *p* < 0.01, and all the data are presented as mean + std dev.

As illustrated by the cadaveric biomechanical testing, marked decreases in the failure load were observed in the 25% defect (−41.1%, *p* = 0.038) and 50% defect groups (−56.8%, *p* = 0.004) when compared with the intact femur (Figure 4A). There was a significant improvement in the failure load after insertion of two 6.5-mm cannulated screws in the 50% bone defect model (+95%, *p* = 0.048), and no significant difference was found when this group was compared to that of the intact femur (Figure 4A). As for stiffness measurements, a similar trend was found among the groups but did not reach statistical significance (Figure 4B). Moreover, as can be seen in Figures 2C1–C4, all of the fractures occurred in the femoral neck region.

### FEA

The maximum displacement of the intact femur, 25% defect model, 50% defect model, and 50% defect + two cannulated screw model was 0.885 mm, 0.940 mm, 0.952 mm, and 0.938 mm, respectively. A significant linear correlation between the FEA stiffness and the stiffness of the cadaveric test (r = 0.957, *p* = 0.04) was observed, suggesting that our static analysis FEA models were valid.

**FIGURE 5:**
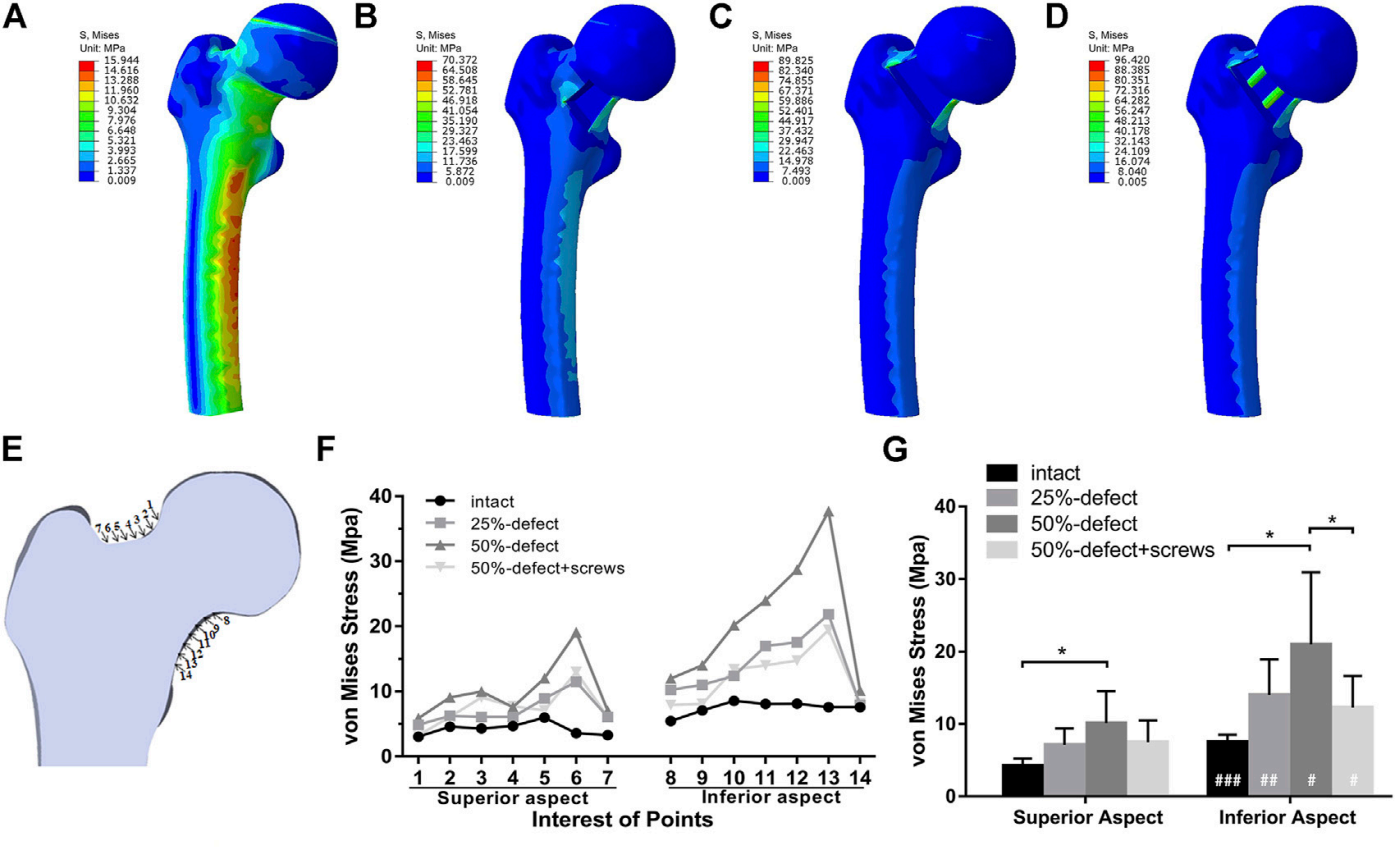
Von Mises stress distribution of the intact femur **(A)**, 25%-defect **(B)**, 50%-defect **(C)**, and 50%-defect + two cannulated screws **(D)** under axial loading with 700 N. **(E)** Measurements of von Mises stress were queried at 14 different points on the femoral neck that lay within a single coronal plane. **(F)** Von Mises stresses of each point on the inferior and superior margin of the femoral neck were obtained. **(G)** Comparison of the mean von Mises stresses between the inferior and superior margin of the femoral neck in the circumstance of the intact femur, 25%-defect, 50%-defect, and 50%-defect + two cannulated screws. * indicated *p* < 0.05 when compared with other groups. ^#^ indicated *p* < 0.05, and ^##^ indicated *p* < 0.01 when compared with the superior margin within each group. All the data are presented as mean + std.

The von Mises stress distributions of the four models are shown in Figures 5A–D. The quantitative von Mises stresses of selected points on the inferior and superior margin of the femoral neck are given in Figures 5E,F. When compared with the intact femur, the 50% defect model showed significantly higher mean von Mises stresses on both the inferior aspect (+140%, *p* = 0.001) and superior aspect (+180%, P<0.001) of the femoral neck (Figure 5G). Insertion of the two screws significantly reduced the mean von Mises stresses on the inferior aspect in the 50% bone defect model (−71%, *p* = 0.043), while no significant difference was found when compared with the control group (Figure 5G). As shown in Figure 5G, there were more significant increases in the von Mises stress measurements on the inferior aspect of the femoral neck than on the superior aspect, in both the 25%-bone defect group (*p* = 0.006) and 50%-bone defect group (*p* = 0.024).

**FIGURE 6:**
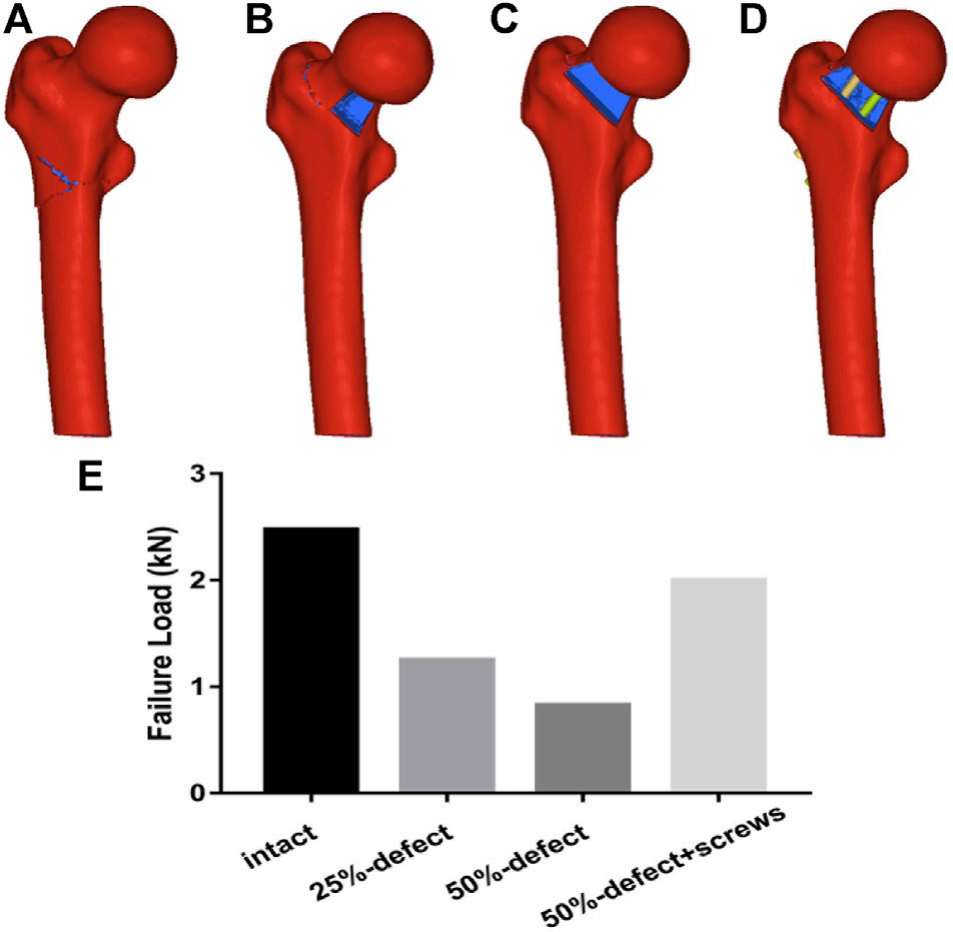
Fracture lines of the four models are shown in Figures 6A–D. Figure 6E. Comparisons of failure load within the four groups are made *via* FEA.

Regarding the failure load testing, the fracture lines of the four models are shown in Figures 6A–D. As illustrated in Figure 6E, there was a marked improvement in the failure load after insertion of two 6.5-mm cannulated screws in the 50% bone defect model. A statistically significant linear correlation of the failure load between FEA and cadaveric tests was also found (r = 0.953, *p* = 0.047).

## Discussion

In the present study, we observed a significant improvement in bone strength after the insertion of two 6.5-mm cannulated screws under the condition of a 50%-bone defect in the femoral neck as determined by cadaveric biomechanical tests and FEA. Similarly, another cadaveric study found that two cannulated screws provided increased axial stiffness and torsional stiffness in an anterior cortical bone defect adult femur sample but without a clear definition of the bone defect area (Dou et al., 2019). To the best of our knowledge, our study represents the first time to investigate the biomechanical stability of two cannulated screws as prophylactic fixation under the condition of an entire anterior cortical bone defect in the femoral neck, which is commonly encountered in adult patients with an aggressive benign femoral neck lesion.

Although no significant difference in the biomechanical properties was found between the two groups, two cannulated screws in a 50% femoral neck cortical bone defect sample did not provide increased biomechanical stability when compared with the intact femur as expected. However, it is important to note that the mean failure load of the two cannulated screw group in our study was approximately 1600 N. During static balance, approximately one-third of a person’s body weight falls on each hip vertically (Çaypınar et al., 2016). Accordingly, 300 N falls vertically on each hip with 600 N on both hips in a 90 kg man during static standing, and 900 N falls on each hip during a one-legged stance. Thus, we suggest that the strength of two cannulated screw fixation was sufficient for full-weight standing and walking, especially in patients with normal body weight. However, our fixation strategy might be insufficient in all circumstances as up to 10 times a person’s body weight can be exerted on the hips during running or intense activities (Çaypınar et al., 2016). Previous clinical studies also reported that no periprosthetic fracture was reported at either 6 months (Singh et al., 2015) or 48 months (Erol et al., 2016) after local curettage and prophylactic fixation using two cannulated screws in benign femoral neck lesion adult patients, with a restriction of intense activities before bone healing. Overall, we suggested that the application of two cannulated screws for prophylactic fixation is feasible for an aggressive benign femoral neck lesion in adult patients, even with an entire anterior cortical bone defect.

In the present study, significant decreases in the biomechanical properties of the femur neck were found in the 25% bone defect and 50% bone defect groups as determined by biomechanical analysis and FEA. A previous cadaver study showed that the failure load significantly decreased when the area of the bone defect increased from 35% to 55% in the femoral neck (Çaypınar et al., 2016). It was also reported that a bone defect located in the femoral neck was associated with worse biomechanical properties in both sideways fall and stance-loading conditions when compared with a bone defect located in the proximal diaphysis (Rajapakse et al., 2019). Clinical studies further confirmed these conclusions as patients with more than 54% bone defect in the femoral neck displayed a higher risk of pathological fractures during follow-up (Jeys et al., 2006; Günther et al., 2007). As we mentioned before, prophylactic fixation for an impending pathologic fracture in patients with painful lesions was associated with higher economic and clinical values when compared with an established fracture (Blank et al., 2016). Consequently, we recommend surgical intervention if the cortical bone is involved, although there remains no guideline for prophylactic bone fixation in patients with benign and tumor-like bone lesions in the femoral neck (Shih et al., 1996; Shin et al., 2013; Erol et al., 2016).

We also observed that there were more significant increases in the von Mises stress measurements on the inferior aspect of the femoral neck than on the superior aspect, in both 25%- and 50%-bone defect groups. Similarly, Benca et al. (2017); Benca et al. (2019) showed stiffness and failure load were significantly lower in specimens with inferior femoral neck lesions than with superior femoral neck lesions. The destruction of cortical bone in the calcar and principal compressive trabeculae might be a reasonable explanation for this observation (Rudman et al., 2006). Thus, we suggest that the site of the lesion in the femoral neck has a potentially large effect on reducing biomechanical properties, and more caution should be applied when lesions are located in the inferior margin of the femoral neck.

The present study has several limitations. First, biomechanical measurements based on FEA and cadaveric tests did not evaluate the strength of two cannulated screw fixation in the circumstance of side-fall as only static standing was simulated. Determining the failure load in a simulated one-legged stance setup cannot be representative for all real-life fracture mechanisms which are inherently different from one patient to another and must account for different pathologies and load inductions (Benca et al., 2017). Nevertheless, the experimental setup and methodology used in this study provided highly reproducible experimental conditions and results comparable to those of previously published work in this field (Çaypınar et al., 2016; Benca et al., 2017; Benca et al., 2019; Dall'Ara et al., 2013). Second, neither cadaveric biomechanical testing nor FEA took into consideration soft tissues including the capsular ligament and periosteum, and this might partially limit the applicability of this testing in *in vivo* situations. Moreover, FEA with homogenous material properties is not based on specimen-specific femur models but on a healthy subject, and the bone defect in our testing was created artificially with only one defined lesion geometry that is consistent with previous studies (Çaypınar et al., 2016; Rajapakse et al., 2019). However, in the clinical setting, bone lesions often affect multiple regions and the cortex. Furthermore, conditions where more than 50% defect of the cortical bone in the femoral neck were not investigated in this study. Nevertheless, as it was reported that under such conditions, there would be insufficient bone to hold the screws, and internal fixation or total hip arthroplasty might be more appropriate approaches in this situation (Zhang et al., 2008). Last, it is necessary to compare the two cannulated screws with other internal fixation methods in order to highlight the advantages of this surgical method and support its clinical application, which we plan to fulfill in future studies.

## Conclusion

Based on cadaveric biomechanical testing and FEA, we found that two 6.5-mm cannulated screws provided sufficient biomechanical strength for prophylactic fixation in adult patients with an aggressive benign femoral neck lesion, where even the entire anterior cortical bone is involved. The current study also provides preliminary evidence for the clinical application of two cannulated screws in the former-mentioned clinical scenario, although the strength of the evidence was limited by the fact that only the circumstance of static standing was mimicked and the FEA was based on one healthy subject. Future biomechanical studies mimicking the circumstance of side-fall and stair climbing, biomechanical testing compared with other internal fixation methods, and long-term clinical follow-up with adequate sample size are needed to further validate our findings.

## Data Availability

The raw data supporting the conclusions of this article will be made available by the authors, without undue reservation.
